# Surface Wettability Tuning of Acrylic Resin Photoresist and Its Aging Performance

**DOI:** 10.3390/s21144866

**Published:** 2021-07-19

**Authors:** Yingying Dou, Fahong Li, Biao Tang, Guofu Zhou

**Affiliations:** 1 Guangdong Provincial Key Laboratory of Optical Information Materials and Technology & Institute of Electronic Paper Displays, South China Academy of Advanced Optoelectronics, South China Normal University, Guangzhou 510006, China; douyingying@scnu.edu.cn (Y.D.); guofu.zhou@m.scnu.edu.cn (G.Z.); 2 Key Laboratory for Polymer Composite & Functional Materials of Ministry of Education, School of Materials Science and Engineering, Sun Yat-sen University, Guangzhou 510006, China; 3 National Center for International Research on Green Optoelectronics, South China Normal University, Guangzhou 510006, China; Frank.Li@scnu.edu.cn; 4 Shenzhen Guohua Optoelectronics Tech. Co. Ltd., Shenzhen 518110, China; 5 Academy of Shenzhen Guohua Optoelectronics, Shenzhen 518110, China

**Keywords:** photoresist, contact angle, wettability tuning, aging

## Abstract

Photoresist is the key material in the fabrication of micropatterns or microstructures. Tuning the surface wettability of photoresist film is a critical consideration in its application of microfluidics. In this work, the surface wettability tuning of acrylic resin photoresist by oxygen plasma or ultra-violet/ozone, and its aging performance in different atmospheres, were systematically studied. The chemical and physical characterizations of the surfaces before and after modification show a dramatic decrease in the C–C group and increase in surface roughness for oxygen plasma treatment, while a decrease of the C–C group was found for the UV/ozone treatment. The above difference in the surface tuning mechanism may explain the stronger hydrophilic modification effect of oxygen plasma. In addition, we found an obvious fading of the wettability tuning effect with an environment-related aging speed, which can also be featured by the decrease of the C–C group. This study demonstrates the dominated chemical and physical changes during surface wettability tuning and its aging process, and provides basis for surface tuning and the applications in microfluidics.

## 1. Introduction

There are special requirements for the wettability of solid surfaces in many fields, including biological process [1,2], membrane [3,4], wastewater treatment [5], metal corrosion resistance [6,7], separation [8], absorbance [9], cleanness [10], sensor [11], etc. For example, a super-hydrophobic film is needed as the barrier to prevent aluminium corrosion [6]; the thermoplastics of a microfluidic device should be hydrophilic enough to avoid the absorption of hydrophobic compounds during the cell-culture process [1]. The pixel wall surface of the electrowetting display needs to be sufficiently hydrophilic to pin the oil/water interface [12]. Many different methods (chemical, physical, and biological ones) are reported to achieve the hydrophilic or hydrophobic modification of solid surfaces [4,13], such as etherification, enzymatic treatment, graft copolymerization, [13] plasma, UV/ozone [13], and hydrophilic or hydrophobic material coating, or deposition [4,14].

Plasma treatment is a powerful way to achieve hydrophilic or hydrophobic properties for polymer surfaces. The plasma atmospheres [15] to achieve hydrophilic property include O_2_ [16,17,18], N_2_ [19], Cl_2_ [20], Ar [21,22], Ar/O_2_ [23], Ar/N_2_ [23], and O_2_/H_2_ [24], while the atmospheres for hydrophobic treatment include CF_4_ [25], SF_6_ [26], and Ar/SF_6_ [23]. Among these atmospheres, oxygen plasma is more frequently used to form hydrophilic polymer surfaces to achieve increased wettability [27] and enhanced adhesion [28] used in microfluidic devices [1,15], biological fields [29], and so on. In exception for traditional polymers [16,21,22,27,28,29], photoresist as a commonly used material in micro-fabrication, highly demands hydrophilic or hydrophobic modifications with a few reports [30,31,32].

Ultra-violet/ozone (UV/ozone) is another commonly used method to achieve hydrophilic activation on polymer surfaces or inorganic material surfaces [33,34], because of the high oxidizing property of UV energy and the ozone [35]. With more UV/ozone exposure time, the surface of many polymers becomes increasingly hydrophilic [36,37]. As plasma and UV/ozone are the two frequently used treatments to achieve superficial hydrophilic property, researchers even pay attention to the comparison between them. In Sham’s review, the UV/ozone treatment was found to be milder than the oxygen plasma, because of the high kinetic energy of oxygen plasma [38]. However, the comparison needs more systemic studies to guide the wettability tuning choices.

When used in microfluidics or other fields, the stability of the hydrophilic polymer surfaces obtained by plasma or UV/ozone treatments should be paid attention to, because of the hydrophobic recovery with time [39,40,41]. As reported, low storage temperature and low humidity is beneficial for the hydrophilic stability [19]. Humidity can affect the aging speed because of the enhanced diffusion of the macromolecules, including the attached oxidized groups, while in dry air, it appears that only functional group reorientation occurs in a layer of the order of a few nanometers [42,43]. Likewise, temperature plays an important role in the aging process because of the faster molecular diffusion at higher temperature [19,44]. Meanwhile, the level of treatments affects the aging process. For example, polymer surface with short UV/ozone exposure time obtains an almost complete hydrophobic recovery, while slow and incomplete recovery for long exposure time samples has been observed [41,45]. During the mechanism studies of the aging process, the migration or the reorientation mechanism is agreed upon by some researchers [46,47,48], which includes the reorientation of superficial hydrophilic groups, away from the surface or the migration of treated polymer chains from the surface to the bulk, and the migration of untreated polymer chains from the bulk to the surface.

Photoresist is an important kind of material to fabricate micropatterns or microstructures [1], which have special wettability requirements in applications. Oxygen plasma was reported to successfully hydrophilically modify the epoxy photoresist SU-8 [30,31,49]. However, oxygen plasma is a strong modification method with an etching effect [49], and may affect the mechanical or other properties of photoresist microstructures. Hence, in this paper, oxygen plasma and UV/ozone treatments were applied on an acrylic resin photoresist surface, to provide the wettability tuning opportunities for different applications. Except for the hydrophilic modification performance, the wettability stability during aging of the acrylic photoresist surface is also compared between oxygen plasma and UV/ozone treatments, while the storing atmosphere includes air and water, which plays an important role in the lifetime of applications. Hence, air/water contact angles were used to characterize the hydrophilic property variation with treatment time and the wettability stability level with storing time. To explore the mechanism of the modification and recovery processes, the chemical and physical changes during the treatments were characterized by X-ray photoelectron spectroscopy (XPS) and atomic force microscope (AFM), respectively.

## 2. Experimental Section

### 2.1. Chemicals and Materials

Indium tin oxide (ITO, 25 nm) coated glass with 0.7 mm thickness and 100 Ω/sq resistance was purchased from Guangdong Jimmy Glass Technology, Ltd. (Foshan, China) and used as the substrate. The acrylic resin photoresist (HN resist) was purchased from Suntific Materials Ltd. (Weifang, China). UP water (18.2 MΩ.cm) was used as the water droplet without further purification, prepared by a Laboratory Water Purification System (Ultrapure UV, Hitech Instruments Co., Ltd., Shanghai, China).

### 2.2. Photoresist Surface Preparation and Modification

The wet photoresist film was coated by a spin coater (SC100, Best Tools, LLC., St. Louis, MI, USA), followed by a pre-baking process on a hot plate (EH20B, LabTech, Beijing, China) with 110 °C and 2.5 min. Exposure process was conducted by using an aligner instrument (URE-2000/35, Institute of Optics and Electronics, Chinese Academy of Sciences, Chengdu, China). After a post-exposure-baking process with 110 °C and 2.5 min on a hot plate, and then hard baking with 150 °C and 30 min in an oven (SFG-01B, Huang Shi Hengfeng Medical Instrument Co. Ltd., Huangshi, China), the preparation of the photoresist film was completed.

Hydrophilic treatment on photoresist surface was carried out by using a reactive ion etching (RIE) machine (ME-6A, Institute of Microelectronics, Chinese Academy of Sciences, Beijing, China) with oxygen plasma (intensity of 100 W) or an ultra-violet/ozone cleaning system (UV/ozone, T10X10/OES/E, UVOCS INC., Lansdale, PA, USA) with the UV light of 254 and 185 nm.

Then, the photoresist surfaces were immersed in air or in water atmosphere to observe the superficial hydrophobic recovery with time. The storing temperature was 22.8 °C ± 0.6 °C with a humidity of 52% ± 3%.

### 2.3. Contact Angle Measurement

A contact angle measurement system (Powereach, Shanghai Zhongchen Digital Technology Apparatus Co., Ltd., Shanghai, China) was used to measure the air/water contact angle. Here, air was chosen as the atmosphere phase, while water was the inner droplet during the measurement. Moreover, the volume of the water droplet for each measurement was 5 μL.

### 2.4. Physical Roughness and Chemical Characterizations

Atomic force microscope (AFM, Cypher, Asylum Research, Santa Barbara, CA, USA) was used to measure the roughness of photoresist surfaces after oxygen plasma or UV/ozone treatment and the roughness after hydrophobic recovery in air (or in water) with time.

X-ray photoelectron spectroscopy (XPS, ESCALAB 250Xi, Thermo Fisher, Waltham, MA, USA) was used to analyze the chemical contents of photoresist surfaces after oxygen plasma or UV/ozone treatment and after hydrophobic recovery in air (or in water) with time. The detection depth was around several nanometers.

## 3. Results and Discussions

### 3.1. Hydrophilic Treatment by Oxygen Plasma or UV/Ozone to Photoresist Surface

The main component of the HN photoresist is acrylic resin with phenol aldehyde modified, so the chemical groups on the surface include –CH_2_–CH_2_– and –COO–, as shown in Figure S1. The –COO– group contributes to its hydrophilic property, which is important for the application in microfluidics. Here, the HN photoresist surface was further hydrophilically treated by oxygen plasma and UV/ozone as below.

#### 3.1.1. Wettability Tuning

**Figure 1:**
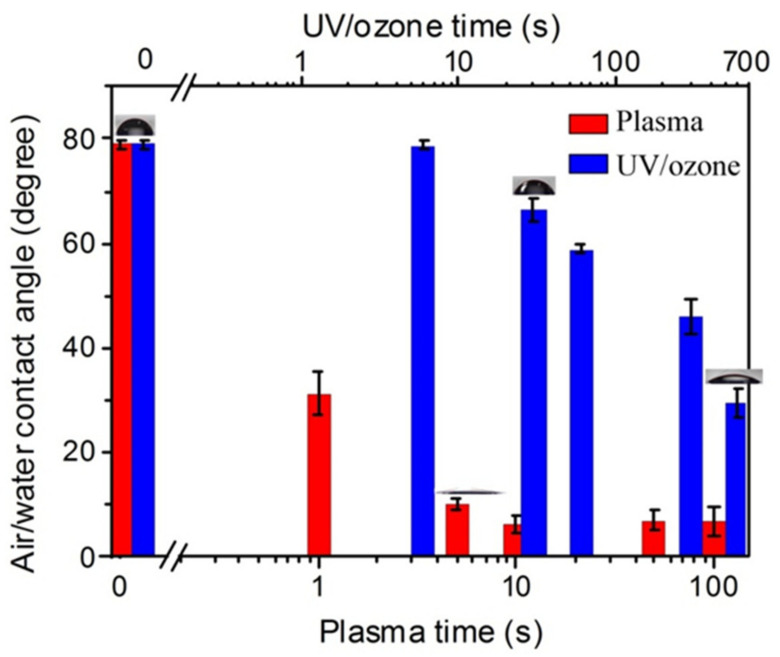
The enhancement of hydrophilic property with increasing oxygen plasma or UV/ozone treatment time on HN photoresist surface. The inserted graphs showing the contact angle of some treatment time.

As shown in Figure 1, after the oxygen plasma treatments with the power of 100 W on the HN photoresist surface, the water contact angle had a big decrease at the initial 10 s, and then kept stable. This indicates enhancement to the hydrophilic property of the surface. On the other hand, UV/ozone treatments with different exposure times also led to a decrease of the water contact angle, which was a continuous decrease with the UV/ozone time, different from the fast decreasing and constant contact angles with oxygen plasma treatments. Moreover, the contact angles, even with 600 s UV/ozone exposing (29°), were still larger than that with 100 s oxygen plasma treatment (7°). These contact angles suggest that the oxygen plasma (100 W) is more powerful than UV/ozone (600 s) for hydrophilic treatments.

#### 3.1.2. Chemical and Physical Characterizations

To explore the cause of the hydrophilic enhancement with oxygen plasma or the UV/ozone, the chemical group ratio of carbon spectra and the superficial physical roughness variations were characterized by XPS and AFM, respectively.

**Figure 2:**
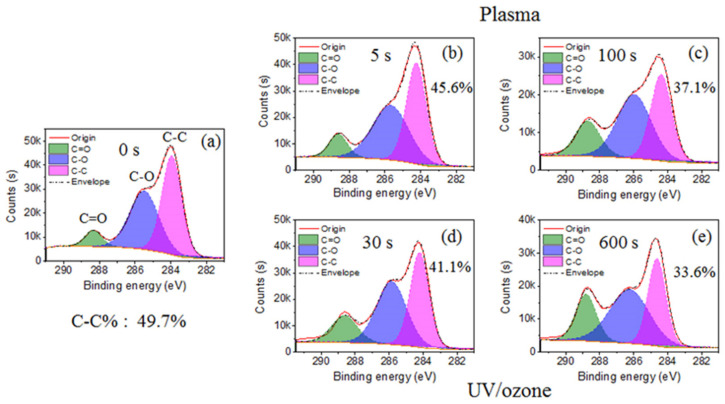
Carbon spectrum from XPS with different plasma or UV/ozone treatment time: (**a**) 0 s, (**b**) plasma 5 s, (**c**) plasma 100 s, (**d**) UV/ozone 30 s, and (**e**) UV/ozone 600 s. There are three peaks: C–C, C–O, and C=O.

The main elements observed in the XPS spectrum of HN photoresist surface (Figure S2) were carbon (C) and oxygen (O). Normally, the chemical groups with oxygen (–C=O, –C–O–, –OH) are more hydrophilic than groups with carbon (–C–C–, –C–H). Hence, the decrease of the C–C group percentage and increase of C–O and C=O groups in carbon spectrum (Figure 2) leads to the improvement of surficial hydrophilic property. During this treatment process, oxygen plasma provided the photoresist surface-active oxygen, which reacted with the HN photoresist surface, resulting in the ratio raise of hydrophilic bonds (C–O (–C–O–C) and C=O (–O–C=O)) (Figure 2b,c). Consequently, the hydrophilic property of HN photoresist surface increased with oxygen plasma treatment, which is consistent with the contact angle variation in Figure 1. After UV/ozone treatments, the percentage of C–C bond (–C–C–) from the carbon spectrum also decreased with UV/ozone time, along with the growth of the percentage of C–O and C=O bonds (Figure 2d,e). The chemical changes above are the important reasons of the contact angle, decreasing with the UV/ozone exposing, i.e., the hydrophilic property enhancement. During the UV/ozone treatment, the ozone atmosphere provided the active oxygen while the UV light (hn) provided the initiation energy [50]. This reaction process led to the increase of the superficial oxygen content, i.e., the increased percentage of C-O and C=O bonds.

**Figure 3:**
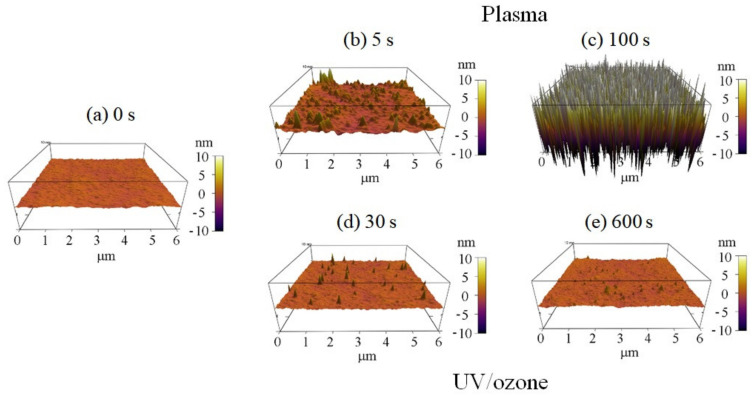
Three-dimensional (3D) AFM observations of the photoresist surfaces with different plasma or UV/ozone treatment time: (**a**) 0 s, (**b**) plasma 5 s, (**c**) plasma 100 s, (**d**) UV/ozone 30 s, and (**e**) UV/ozone 600 s.

The surface roughness of photoresist greatly increased with the oxygen plasma time observed by AFM (Figure 3). The HN photoresist surface without any treatment had a small roughness of 0.35 nm (Figure 3a). Meanwhile, with short oxygen plasma exposure (5 s), the surface roughness increased to 0.83 nm with several peaks (Figure 3b). Increasing the plasma exposure time further to 100 s, the surface roughness increased greatly to 6.65 nm with a large number of peaks (Figure 3c), which is much higher than the peaks in Figure 3b. The surface roughness increase with plasma time was the other factor affecting the superficial hydrophilic property. Meanwhile, there was no big change of the superficial physical roughness for different UV/ozone time (Figure 3). Some inhomogeneous peaks are observed in the 3D graphs (Figure 3d,e), affecting the roughness, for a few, because of their small quantities.

Overall, the water contact angle of the HN photoresist surface reduced and then kept constant with oxygen plasma exposing time (Figure 1), due to the combined influence of the chemical content changing, due to the reaction between active oxygen and photoresist polymer and the physical roughness variations, due to the physical etching by plasma. Meanwhile, with the UV/ozone treatment, the hydrophilic property of HN photoresist surface also enhanced with treatment time (Figure 1), although weaker than oxygen plasma. The main cause of the hydrophilic modification of the UV/ozone was due to the superficial chemical group changes, other than the superficial physical roughness changes. In summary, oxygen plasma and UV/ozone is useful for the hydrophilic modification of the acrylic HN photoresist surface. Meanwhile, be careful of the transmittance decrease (Figure S3) due to strong UV/ozone treatment, which affects the film optical performance.

### 3.2. Wettability Aging with Time in Air or in Water Atmosphere

#### 3.2.1. Contact Angle Variation during Aging

**Figure 4:**
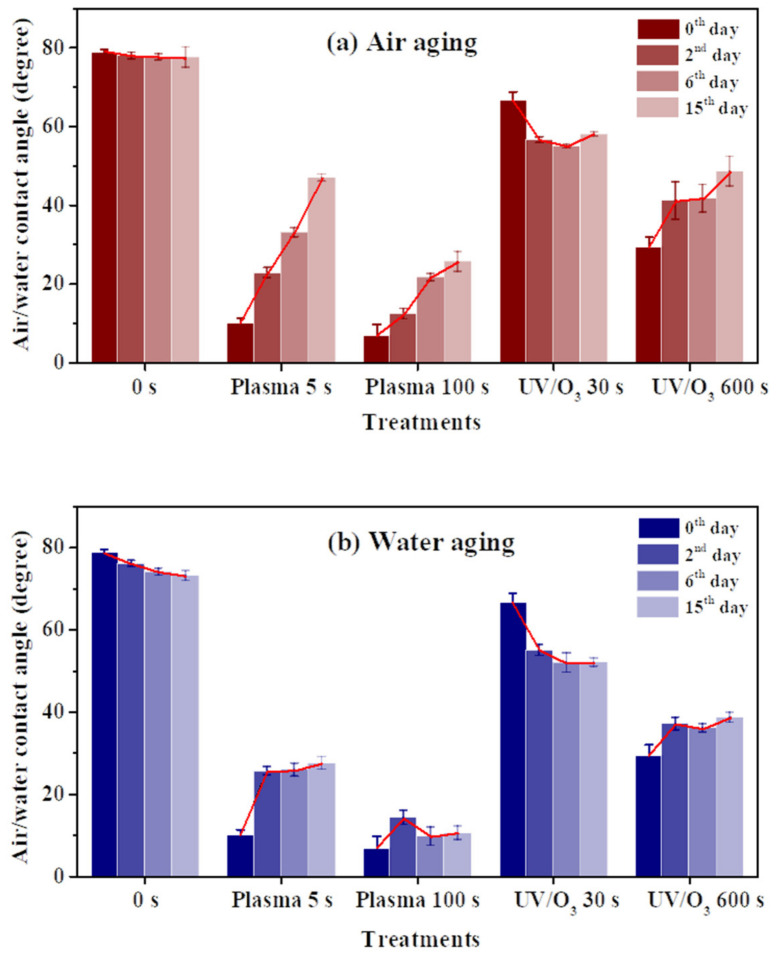
The water contact angle variations of HN photoresist surface after oxygen plasma treatments or UV/ozone (UV/O_3_) treatments with aging. The storing atmosphere is air (**a**) or water (**b**).

**Figure 5:**
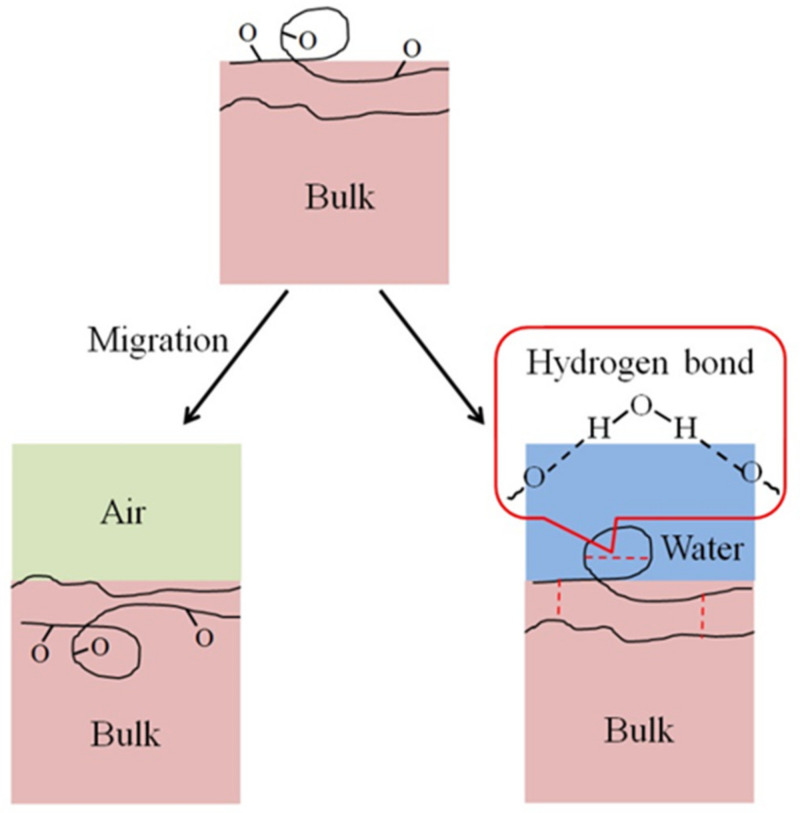
Scheme of the migration or reorientation process between surface and bulk, easy in air and difficult in water because of the hydrogen bond formation. The hydrogen bonds include those in one photoresist polymer chain and between two polymer chains.

A contact angle increase of the HN photoresist surface was observed after the oxygen plasma hydrophilic treatment storing in air or in water, as shown in Figure 4a,b. When the storing atmosphere was air, the contact angle kept increasing in the experimental time (15 days), while the contact angle only had an obvious increase at the beginning and then almost remained constant in water. Moreover, the final contact angles, at the 15th day for samples in water, were much lower than those stored in air for different extents of oxygen plasma treatments. This indicates that water is beneficial for long-term wettability stability of HN photoresist surface. In water atmosphere storing, the formation of hydrogen-bonds (–O---H–OH, or –O---H–O–H---O–) [51,52] between the –COO– groups and water blocked the HN photoresist polymer chains for the further migration or reorientation [53] between the surface and the bulk, as illustrated in Figure 5. 

The wettability of photoresist surfaces after UV/ozone treatments performed differently from the oxygen plasma one. As shown in Figure 4a,b, the contact angles increased during aging for the HN photoresist surfaces with strong (600 s) UV/ozone treatment, and decreased for samples with weak (30 s) treatments. The energy of UV light activated the ozone molecules to activated oxygen, and then the reaction between active oxygen and HN photoresist surface occurred. Generally, during the aging process, the hydrophilic chemical groups migrate or reorient from the surface to the bulk, leading to the contact angle increasing. The explanation of the contact angle decreasing with weak UV/ozone treatment will be discussed in the next section.

#### 3.2.2. Chemical and Physical Analysis

Plasma treatment samples and a strong UV/ozone treatment sample has the contact angle increase, while the contact angle of the weak UV/ozone sample decreases during aging (Figure 4), especially in 2 days. To explore this aging mechanism, chemical group ratio changes and physical roughness changes are analyzed in this section. 

**Figure 6:**
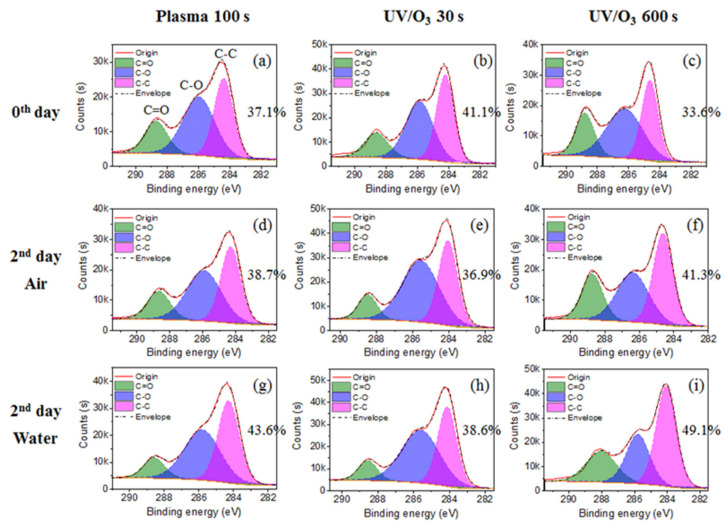
The chemical group percentage changes from carbon spectrum before aging (**a**–**c**) and after 2 days aging in air (**d**–**f**) or in water (**g**–**i**). The chemical groups include C–C, C–O, and C=O. The treatments include plasma 100 s, UV/O_3_ 30 s, and UV/O_3_ 600 s.

The C–C group percentage increased for the plasma 100 s sample and the UV/ozone 600 s sample, and decreased for the UV/ozone 30 s sample (Figure 6), which was the reason for the irregularly contact angle decreasing of the UV/ozone 30 s sample. With the decrease of the C–C group percentage, the C–O group percentage increases, which may be due to some unfinished reaction between the photoresist polymer and activated oxygen by the UV light in a short 30 s treatment time. Meanwhile, the C–C group percentage increase with the C–O group percentage decrease for the plasma 100 s and UV/ozone 600 s samples, as desired, was due to the normal migration or reorientation mechanism during aging. This indicates that more hydrophobic groups (C–C) migrate or reorientate to the surface and more hydrophilic groups (C–O) migrate or reorientate to the bulk.

**Figure 7:**
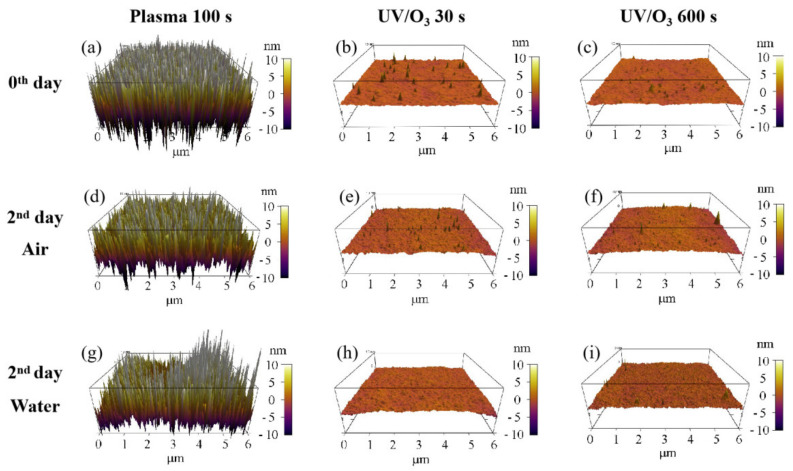
Roughness changes before aging (**a**–**c**) and after 2 days aging in air (**d**–**f**) or in water (**g**–**i**). The treatments include plasma 100 s, UV/O_3_ 30 s, and UV/O_3_ 600 s.

The migration or reorientation process during the 2-day aging led to the decrease of roughness for the plasma 100 s sample, with no big influence on UV/ozone samples (Figure 7). Because of the nanostructures’ formation on the surface of the plasma 100 s sample (Figure 3c), the roughness influence on the water contact angle was complex. However, the final result was that the water contact angle increased after 2 days of aging, so we believe that the combination of chemical content and physical roughness on the surface of the plasma 100 s sample leads to the water contact angle increase—the hydrophilic instability. Meanwhile, only the chemical content change results in water contact angle variations for the UV/ozone samples. 

## 4. Conclusions

The acrylic photoresist surface has hydrophilic enhancement after the wettability modification by oxygen plasma or the UV/ozone, showing a decrease of the air/water contact angle. The C–C percentage in the carbon spectrum of XPS observations decreases after oxygen plasma or UV/ozone treatment, indicating the chemical content variation, which is the chemical cause of superficial hydrophilic enhancement. Surface roughness increases after oxygen plasma treatment and there is no big change after the UV/ozone treatment, which is beneficial for the hydrophilic enhancement of surfaces after plasma treatments. Water contact angles increase during aging except for the short UV/ozone exposure (30 s), indicating the bad wettability stability after oxygen plasma or UV/ozone treatments. Surfaces stored in the air atmosphere have a contact angle increase during aging, while contact angle increase stops on the 2nd day for surfaces stored in water, due to the hydrogen bond forming with water molecules, which has more energy and is difficult to break for migration. This indicates water is better for wettability stability of the acrylic photoresist surface after plasma or UV/ozone modifications. The C–C group percentage in the carbon spectrum variation after 2 days is the main reason for the above contact angle variations, such as the water contact angle increase for the UV/ozone 30 s sample. Meanwhile, roughness has no change for samples with UV/ozone treatments, and shows (nearly) no influence on the contact angle recovery. 

## Data Availability

The data presented in this study are available on request from the corresponding author.

## Supplementary Materials

The following are available online at https://www.mdpi.com/article/10.3390/s21144866/s1, Figure S1. FT-IR spectrum of HN photoresist. (3031.6 cm^−1^, C-H (benzene series); 2925.0 cm^−1^, C-H (-CH_2_); 1725.4 cm^−1^, C=O (-COOR); 1634.8 cm^−1^ and 1616.5 cm^−1^, C=C; 1476.2 cm^−1^, C-H(-CH_2_); 1407.2 cm^−1^, C-H(-CH_2_-C=O); 1245.8 cm^−1^ and 1188.0 cm^−1^, C-O-C (-COOR); 809.0 cm^−1^ and 660.0 cm^−1^, C-H (benzene series, from the phenol aldehyde modification part to the resin); Figure S2. The survey spectrum from XPS with different oxygen plasma or UV/ozone treatment time (0 s, plasma 5 s, plasma 100 s, UV/ozone 30 s, and UV/ozone 600 s). The carbon (C) and oxygen (O) peaks are pointed out; Figure S3. Transmittance curve (**a**) and the photos (**b**) of samples without treatment (0 s) or after oxygen plasma treatment (5 s or 100 s) or UV/ozone treatment (30 s or 600 s). The wavelength range is 400–800 nm, and the reference is air.

## References

[1] Van Midwoud P.M., Janse A., Merema M.T., Groothuis G.M., Verpoorte E. (2012). Comparison of biocompatibility and adsorption properties of different plastics for advanced microfluidic cell and tissue culture models. Anal. Chem..

[2] Matsui H., Oaki Y., Imai H. (2016). Surface-functionalized hydrophilic monolayer of titanate and its application for dopamine detection. Chem. Commun..

[3] Wang K., Hou D., Wang J., Wang Z., Tian B., Liang P. (2018). Hydrophilic surface coating on hydrophobic PTFE membrane for robust anti-oil-fouling membrane distillation. Appl. Surf. Sci..

[4] Kang G.D., Cao Y.M. (2014). Application and modification of poly(vinylidene fluoride) (PVDF) membranes—A review. J. Membr. Sci..

[5] Zinadini S., Gholami F. (2016). Preparation and characterization of high flux PES nanofiltration membrane using hydrophilic nanoparticles by phase inversion method for application in advanced wastewater treatment. J. Appl. Res. Water Wastewater.

[6] Lu Z., Wang P., Zhang D. (2015). Super-hydrophobic film fabricated on aluminium surface as a barrier to atmospheric corrosion in a marine environment. Corros. Sci..

[7] Syed J.A., Tang S., Meng X. (2017). Super-hydrophobic multilayer coatings with layer number tuned swapping in surface wettability and redox catalytic anti-corrosion application. Sci. Rep..

[8] Chang Q., Zhou J.E., Wang Y., Liang J., Zhang X., Cerneaux S., Wang X., Zhu Z., Dong Y. (2014). Application of ceramic microfiltration membrane modified by nano-TiO_2_ coating in separation of a stable oil-in-water emulsion. J. Membr. Sci..

[9] Zhong H., Jiang Y., Zeng G., Liu Z., Liu L., Liu Y., Yang X., Lai M., He Y. (2015). Effect of low-concentration rhamnolipid on adsorption of Pseudomonas aeruginosa ATCC 9027 on hydrophilic and hydrophobic surfaces. J. Hazard. Mater..

[10] Oribayo O., Pan Q., Feng X., Rempel G.L. (2017). Hydrophobic Surface Modification of FMSS and its Application as Effective Sorbents for Oil Spill Clean-ups and Recovery. Aiche J..

[11] Zhao L., Yang J., Ye H., Zhao F., Zeng B. (2017). Preparation of hydrophilic surface-imprinted ionic liquid polymer on multi-walled carbon nanotubes for the sensitive electrochemical determination of imidacloprid. RSC Adv..

[12] Roques-Carmes T., Palmier S., Hayes R.A., Schlangen L.J.M. (2005). The effect of the oil/water interfacial tension on electrowetting driven fluid motion. Colloids Surf. A.

[13] Ali A., Shaker K., Nawab Y., Jabbar M., Hussain T., Militky J., Baheti V. (2016). Hydrophobic treatment of natural fibers and their composites—A review. J. Ind. Text..

[14] Trantidou T., Elani Y., Parsons E., Ces O. (2017). Hydrophilic surface modification of PDMS for droplet microfluidics using a simple, quick, and robust method via PVA deposition. Microsyst. Nanoeng..

[15] Palumbo F., Lo Porto C., Favia P. (2019). Plasma Nano-Texturing of Polymers for Wettability Control: Why, What and How. Coatings.

[16] Zhu D., Lu X., Lu Q. (2014). Electrically conductive PEDOT coating with self-healing superhydrophobicity. Langmuir.

[17] Jaleh B., Etivand E.S., Mohazzab B.F., Nasrollahzadeh M., Varma R.S. (2019). Improving Wettability: Deposition of TiO_2_ Nanoparticles on the O_2_ Plasma Activated Polypropylene Membrane. Int. J. Mol. Sci..

[18] Rodrigues M.M., Fontoura C.P., Garcia C.S.C., Martins S.T., Henriques J.A.P., Figueroa C.A., Roesch-Ely M., Aguzzoli C. (2019). Investigation of plasma treatment on UHMWPE surfaces: Impact on physicochemical properties, sterilization and fibroblastic adhesion. Mater. Sci. Eng. C Mater. Biol. Appl..

[19] Chen F., Liu J., Yao C., Huang S., Song J., Sun J., Xu W., Liu X. (2016). Stability of plasma treated superhydrophobic surfaces under different ambient conditions. J. Colloid Interface Sci..

[20] Jung S.-G., Choi K.B., Park C.H., Shim Y.S., Park C.H., Park Y.W., Ju B.-K. (2019). Effects of Cl_2_ plasma treatment on stability, wettability, and electrical properties of ITO for OLEDs. Opt. Mater..

[21] Deynse A.V., Cools P., Leys C., Morent R., Geyter N.D. (2015). Surface modification of polyethylene in an argon atmospheric pressure plasma jet. Surf. Coat. Technol..

[22] Correia D.M., Nunes-Pereira J., Alikin D., Kholkin A.L., Carabineiro S.A.C., Rebouta L., Rodrigues M.S., Vaz F., Costa C.M., Lanceros-Méndez S. (2019). Surface wettability modification of poly(vinylidene fluoride) and copolymer films and membranes by plasma treatment. Polymer.

[23] Lock E.H., Petrovykh D.Y., Mack P., Carney T., White R.G., Walton S.G., Fernsler R.F. (2010). Surface composition, chemistry, and structure of polystyrene modified by electron-beam-generated plasma. Langmuir.

[24] Lin C.H., Tsai M.S., Chen W.T., Hong Y.Z., Chien P.Y., Huang C.H., Woon W.Y., Lin C.T. (2019). A low-damage plasma surface modification method of stacked graphene bilayers for configurable wettability and electrical properties. Nanotechnology.

[25] Peng S., Ma Y. (2017). Fabrication of hydrophilic and oil-repellent surface via CF_4_ plasma treatment. Mater. Des..

[26] Saloum S., Shaker S.A., Alkafri M.N., Obaid A., Hussin R. (2020). Hydrogenated Silicon Carbonitride Thin Film Nanostructuring Using SF_6_ Plasma: Structural and Optical Analysis. Silicon.

[27] Holc M., Zaplotnik R., Mozetic M., Vesel A. (2018). Surface Modification and Aging of Polyacrylonitrile Butadiene Styrene Polymer Induced by Treatment in RF Oxygen Plasma. IEEE Trans. Plasma Sci..

[28] Juárez-Moreno J.A., Ávila-Ortega A., Oliva A.I., Avilés F., Cauich-Rodríguez J.V. (2015). Effect of wettability and surface roughness on the adhesion properties of collagen on PDMS films treated by capacitively coupled oxygen plasma. Appl. Surf. Sci..

[29] Alves P., Cardoso R., Correia T.R., Antunes B.P., Correia I.J., Ferreira P. (2014). Surface modification of polyurethane films by plasma and ultraviolet light to improve haemocompatibility for artificial heart valves. Colloids Surf. B.

[30] Walther F., Davydovskaya P., Zürcher S., Kaiser M., Herberg H., Gigler A.M., Stark R.W. (2007). Stability of the hydrophilic behavior of oxygen plasma activated SU-8. J. Micromech. Microeng..

[31] Jokinen V., Suvanto P., Franssila S. (2012). Oxygen and nitrogen plasma hydrophilization and hydrophobic recovery of polymers. Biomicrofluidics.

[32] Shui L., Hayes R.A., Jin M., Zhang X., Bai P., van den Berg A., Zhou G. (2014). Microfluidics for electronic paper-like displays. Lab Chip.

[33] Mulyana Y., Uenuma M., Ishikawa Y., Uraoka Y. (2014). Reversible Oxidation of Graphene Through Ultraviolet/Ozone Treatment and Its Nonthermal Reduction through Ultraviolet Irradiation. J. Phys. Chem. C.

[34] Nguyen P.Q.M., Yeo L.-P., Lok B.-K., Lam Y.-C. (2014). Patterned surface with controllable wettability for inkjet printing of flexible printed electronics. Acs Appl. Mater. Interfaces.

[35] Šojić D., Despotović V., Orčić D., Szabó E., Arany E., Armaković S., Illés E., Gajda-Schrantz K., Dombi A., Alapi T. (2012). Degradation of thiamethoxam and metoprolol by UV, O_3_ and UV/O_3_ hybrid processes: Kinetics, degradation intermediates and toxicity. J. Hydrol..

[36] Maeda H., Yamagishi R., Ishida E.H., Kasuga T. (2019). Wettability and dynamics of water droplet on a snail shell. J. Colloid Interface Sci..

[37] Lee J.-Y., Park C.-Y., Moon S.-Y., Choi J.-H., Chang B.-J., Kim J.-H. (2019). Surface-attached brush-type CO_2_-philic poly(PEGMA)/PSf composite membranes by UV/ozone-induced graft polymerization: Fabrication, characterization, and gas separation properties. J. Membr. Sci..

[38] Sham M.L., Li J., Ma P.C., Kim J.-K. (2009). Cleaning and Functionalization of Polymer Surfaces and Nanoscale Carbon Fillers by UV/Ozone Treatment: A Review. J. Compos. Mater..

[39] Pascual M., Kerdraon M., Rezard Q., Jullien M.C., Champougny L. (2019). Wettability patterning in microfluidic devices using thermally-enhanced hydrophobic recovery of PDMS. Soft Matter.

[40] Lin W.C., Razali N.A.M. (2019). Temporary Wettability Tuning of PCL/PDMS Micro Pattern Using the Plasma Treatments. Materials.

[41] Hillborg H., Tomczak N., Olàh A., Schönherr H., Vancso G.J. (2004). Nanoscale hydrophobic recovery: A chemical force microscopy study of UV/ozone-treated cross-linked poly(dimethylsiloxane). Langmuir.

[42] Tsougeni K., Vourdas N., Tserepi A., Gogolides E., Cardinaud C. (2009). Mechanisms of oxygen plasma nanotexturing of organic polymer surfaces: From stable super hydrophilic to super hydrophobic surfaces. Langmuir.

[43] Dupont-Gillain C.C., Adriaensen Y., Derclaye S., Rouxhet P.G. (2000). Plasma-Oxidized Polystyrene:  Wetting Properties and Surface Reconstruction. Langmuir.

[44] Mortazavi M., Nosonovsky M. (2012). A model for diffusion-driven hydrophobic recovery in plasma treated polymers. Appl. Surf. Sci..

[45] Bilgin S., Isik M., Yilgor E., Yilgor I. (2013). Hydrophilization of silicone–urea copolymer surfaces by UV/ozone: Influence of PDMS molecular weight on surface oxidation and hydrophobic recovery. Polymer.

[46] Owen M.J., Smith P.J. (1997). Plasma treatment of polydimethylsiloxane. J. Adhes. Sci. Technol..

[47] Oláh A., Hillborg H., Vancso G.J. (2005). Hydrophobic recovery of UV/ozone treated poly(dimethylsiloxane): Adhesion studies by contact mechanics and mechanism of surface modification. Appl. Surf. Sci..

[48] O’Connell C., Sherlock R., Ball M.D., Aszalós-Kiss B., Prendergast U., Glynn T.J. (2009). Investigation of the hydrophobic recovery of various polymeric biomaterials after 172 nm UV treatment using contact angle, surface free energy and XPS measurements. Appl. Surf. Sci..

[49] Anbumani S., Silva A.M., Roggero U.F.S., Silva A.M.P.A., Hernández-Figueroa H.E., Cotta M.A. (2021). Oxygen plasma-enhanced covalent biomolecule immobilization on SU-8 thin films: A stable and homogenous surface biofunctionalization strategy. Appl. Surf. Sci..

[50] Kearney P.C., Ruth J.M., Zeng Q., Mazzocchi P. (1985). UV-ozonation of paraquat. J. Agric. Food Chem..

[51] Sreenivasulu B., Vittal J.J. (2004). Helix inside a Helix: Encapsulation of Hydrogen-Bonded Water Molecules in a Staircase Coordination Polymer. Angew. Chem. Int. Ed..

[52] Dormidontova E.E. (2002). Role of Competitive PEO−Water and Water−Water Hydrogen Bonding in Aqueous Solution PEO Behavior. Macromolecules.

[53] Tamai Y., Tanaka H., Nakanishi K. (1996). Molecular Dynamics Study of Polymer−Water Interaction in Hydrogels. 1. Hydrogen-Bond Structure. Macromolecules.

