# Supplementary material for: Surface Wettability Tuning of Acrylic Resin Photoresist and Its Aging Performance
Source: Sensors (Basel). 2021 Jul 19;21(14):4866. doi: 10.3390/s21144866 (PMC8309867; doi:10.3390/s21144866)
Supplement: Supplementary file 1 [file sensors-21-04866-s001.zip › sensors-1284689-supplementary.pdf]

Chemical Characterization of HN Photoresist

The chemical groups were characterized by flourier transform infrared spectrometer (FT-IR, Thermo Scientific Nicolet iS50, Thermo-Nicolet, Waltham, MA, USA) with the support of the ATP appendix (iS50 ATP). The fabricated HN photoresist film on glass was characterized by the FT-IR machine with air as the reference.

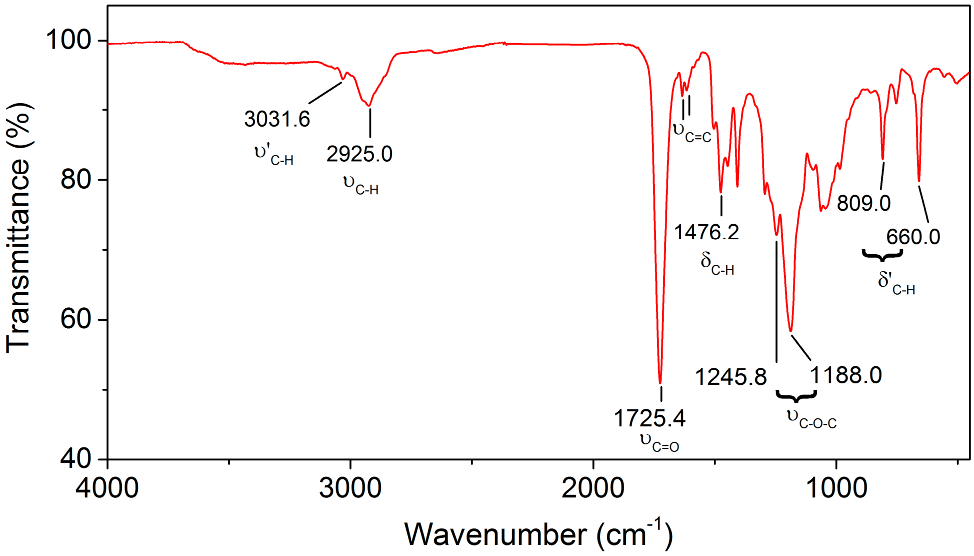

Figure S1. FT-IR spectrum of HN photoresist. (3031.6 cm^{-1}, C-H (benzene series); 2925.0 cm^{-1}, C-H (-CH_2); 1725.4 cm^{-1}, C=O (-COOR); 1634.8 cm^{-1} and 1616.5 cm^{-1}, C=C; 1476.2 cm^{-1}, C-H(-CH_2); 1407.2 cm^{-1}, C-H(-CH_2-C=O); 1245.8 cm^{-1} and 1188.0 cm^{-1}, C-O-C (-COOR); 809.0 cm^{-1} and 660.0 cm^{-1}, C-H (benzene series, from the phenol aldehyde modification part to the resin).

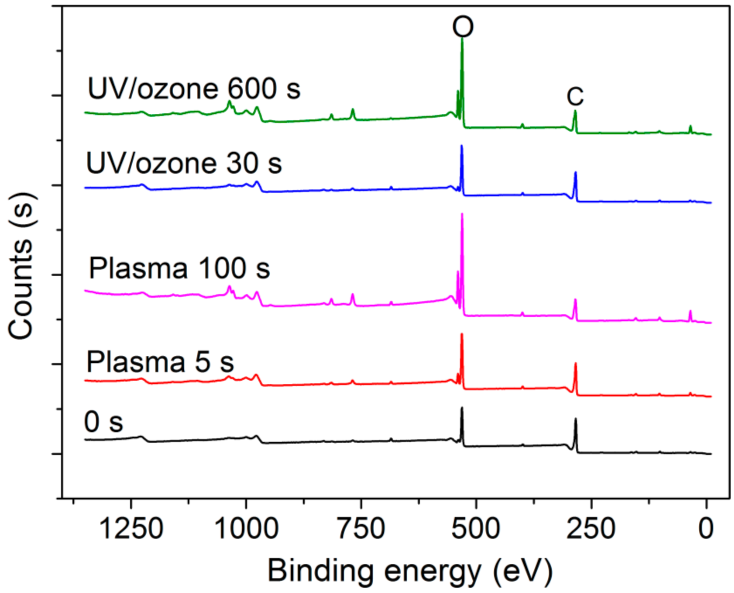

Figure S2. The survey spectrum from XPS with different oxygen plasma or UV/ozone treatment time (0 s, plasma 5 s, plasma 100 s, UV/ozone 30 s, and UV/ozone 600 s). The carbon (C) and oxygen (O) peaks are pointed out.

Optical Performance of HN Photoresist Film before or after Hydrophilic Treatment

The transmittance was used to characterize the optical performance before or after oxygen plasma or UV/ozone treatment measured by a UV-vis spectrophotometer (V-630, Jasco, Japan) with a wavelength range from 400 nm to 800 nm and reference of air. Meanwhile, photos were taken by a camera (EOS 80D, Nikon, Japan).

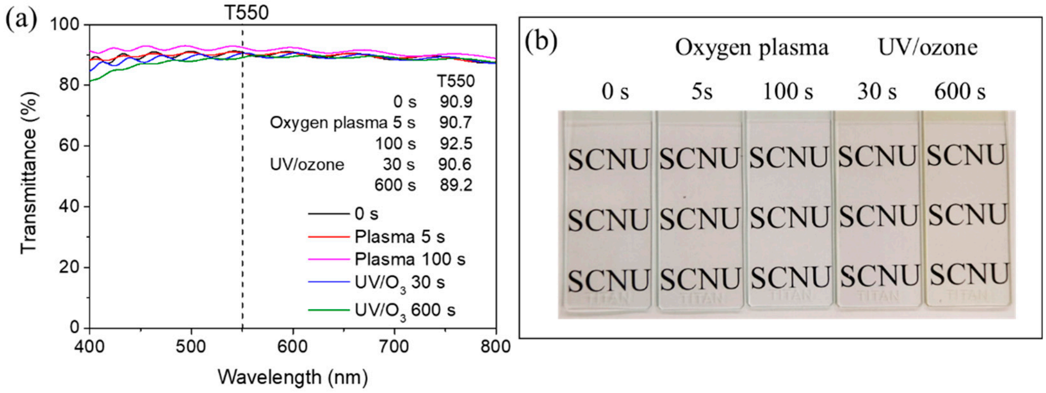

Figure S3. Transmittance curve (**a**) and the photos (**b**) of samples without treatment (0 s) or after oxygen plasma treatment (5 s or 100 s) or UV/ozone treatment (30 s or 600 s). The wavelength range is 400–800 nm, and the reference is air.
